# Improvement of Advanced Sample Preparation Systems for the Determination of Trace Ni in Seawater by Electro-Membranes

**DOI:** 10.3390/membranes13020152

**Published:** 2023-01-24

**Authors:** Juan J. Pinto, Carolina Mendiguchía, José A. López-López, Mabel Martín-Barata, Macarena Silva, Carlos Moreno

**Affiliations:** Department of Analytical Chemistry, Faculty of Marine and Environmental Sciences, University of Cadiz, 11510 Puerto Real, Spain

**Keywords:** electro-membrane extraction, nickel, seawater, hollow fiber liquid phase micro-extraction, di-2-ethylhexyl phosphoric acid (DEHPA)

## Abstract

Due to its important environmental role, the analysis of trace metals in natural waters is attracting increasing attention; consequently, faster and more accurate analytical methods are now needed to reach even lower limits of detection. In this work, we propose the use of electro-membrane extraction (EME) to improve analytical methods based on hollow fiber liquid phase micro-extraction (HFLPME). Specifically, an EME-based method for the determination of trace Ni in seawater has been developed, using an HFLPME system with di-2-ethylhexyl phosphoric acid (DEHPA) in kerosene as a chemical carrier, followed by instrumental determination by graphite furnace atomic absorption spectroscopy (GFAAS). Under optimum conditions, Ni was pre-concentrated 180 ± 17 times after 15 min, using sample pH = 5.5, the concentration of DEHPA 0.9 M in the liquid membranes, and 1.9 M HNO_3_ in the acceptor solution, as well as an electric potential of 25 V with the sample being stirred at 500 rpm. When compared with other HFLPME systems for pre-concentration of trace Ni in seawater in the absence of electric potential, the enrichment factor was improved 2.2 times, while the time of extraction was reduced an 89%. The limit of detection of the new method was 23.3 ng L^−1^, and both its applicability and accuracy were successfully evaluated by analyzing Ni concentration in a seawater-certified reference material (BCR-403), showing the reliability of EME for sample preparation in the determination of trace metals in marine water samples.

## 1. Introduction

Nickel is an essential metal whose concentration in seawater is often at the level of μg L^−1^ or sub-μg L^−1^. However, it tends to accumulate in aquatic organisms, exceeding the toxicity of Pb or Sn, among others [1]. Due to the environmental implications of Ni, it has been considered a priority substance to be controlled in natural waters by the European Union [2], which established the maximum tolerable Ni concentration in 2 μg L^−1^, having been reported in the range of 0.3–3.7 μg L^−1^ in UE surface seawaters [3].

Ni determination in seawater samples requires sample preparation prior to instrumental analysis. In this regard, liquid micro-extraction methods for sample preparation with analytical purposes have been popularized in the last few years as a green alternative to traditional methods [4]. In particular, hollow fiber liquid phase micro-extraction (HFLPME) has been widely applied in the case of organic analysis as well as for metals determination due to its higher stability and easier handling if compared to other micro-extraction techniques [5]. Notwithstanding, there are some aspects that must be improved until this methodology can be applied as a well-established alternative for routine analysis. One of these drawbacks is the long time needed for the experiment often required for pre-concentration, which is particularly important in the case of metals [6].

Additionally, the existence of micro-extraction systems suitable for the pre-concentration of metals from seawater samples is limited by the lack of selective reagents for metal complexation, as well as by a saline sample matrix [7]. Consequently, even when effective reagents are found, a long time is required for the experiments to get enough pre-concentration [8].

To overcome these drawbacks, the use of electrically enhanced hollow fiber liquid phase micro-extraction, in the form of electro-membranes, could be an alternative to promote the migration of ions from the sample into the acceptor solution [9]. This way, enough enrichment factors could be achieved in shorter times.

Electro-membrane extraction (EME) is a rising methodology introduced by Pedersen-Bjergaard et al. in 2006 [10]. EME can be described as an evolution of three phases of liquid phase micro-extraction (3-HFLPME) [8]. In a regular 3 HFLPME, the fiber, containing an aqueous acceptor solution, is immersed in the sample, and the transport of the metal takes place through an organic solution containing a carrier located in the micro-pores of the fiber wall [11]. However, in the case of electro-membranes, an electric potential is applied between the sample and the acceptor solution using two platinum wires as electrodes [10]. Among the advantages of EME over other HFLPME methods is the fact that extraction times can be significantly reduced due to the acceleration in the transport of the analyte, and the amount of sample and acceptor solution can be reduced [12]. Despite the fact that EME has been extensively developed for organic compounds, the number of applications for the pre-concentration of metals is limited.

As recently reviewed, EME has been used for the pre-concentration of divalent ions (Pb^2+^, Ni^2+^, Mn^2+^, Cd^2+^, Cu^2+^, Co^2+^, Zn^2+^, Hg^2+^) in soft water, Th and Ag [6,13,14,15]. However, the reported limit of detection is not enough to measure normally found concentrations of Ni in seawater, and the system does not take into account the influence of a saline matrix. When evaluated, major ions present in seawater (Na^+^, K^+^, Ca^2+^, Mg^2+)^ have been shown to have the same effect in EME as in regular HFLPME, so EME has not been proven to improve the selectivity of the extraction. However, extraction times to reach enough pre-concentration of the metals were dramatically decreased [14]. Additionally, As and Cr oxoanions have also been determined in soft water using electro-membranes [16,17,18], which have also been used for separation of Cr^6+^ and Cr^3+^ [19]. Moreover, Ag pre-concentration using tri-isobutylphosphine sulfide has been enhanced using EME. However, to date, the application to complex matrices as seawater samples is limited to the determination of U [20].

This work is based on previous studies on sample preparation for Ni detection in seawater by HFLPME [21], with the aim of improving the most limiting operational aspect of HFLPME, which is the slow pre-concentration rate. In this case, an electro-membrane has been developed to improve the applicability of HFLPME, with a significant time reduction and efficiency enhancement.

## 2. Materials and Methods

### 2.1. Reagents and Solutions

All reagents were analytical-reagent grade unless otherwise stated. Acetic acid (99.7%), kerosene (97.5%), sodium chloride (99%), potassium chloride (99%), boric acid (99%), and sodium hydroxide (99.0%) were supplied by Panreac (Barcelona, Spain). The extractant di-2-ethylhexyl phosphoric acid (97%) was obtained from Aldrich (Steinheim, Germany). Calcium chloride (99%), magnesium chloride (99.5%), sodium dihydrocarbonate (99%), potassium bromide (99.5%), and sodium sulfate (99%) were obtained from Merck (Darmstadt, Germany). Analytical grade nitric acid (65%) from Scharlau (Barcelona, Spain) was used during optimization experiments, while for application, real samples of suprapur nitric acid (65%) were obtained from Merck (Darmstadt, Germany) and employed. Pure water obtained by a Millipore Quantum Ultrapure water supplier (Bedford, MA, USA) was used exclusively. Argon for atomic spectrometry was obtained from Air Liquide (Madrid, Spain).

Aqueous solutions of nickel were prepared from a 1000 mg L^−1^ standard solution obtained from Scharlau (Barcelona, Spain), and synthetic seawater samples were prepared according to the protocol described by Grasshoff et al., so main ions present in seawater samples, which could interfere in Ni transport, were included at their average concentration level in real samples [22].

Certified reference material BCR-403 for analysis of metals in seawater was obtained from the Institute of Reference Materials and Measurements in the Joint Research Centre (Geel, Belgium).

### 2.2. Equipment

Polypropylene porous hollow fiber Accurel^®^ PP S6/2 from 3 M-Membrana (Wuppertal, Germany) with 1800 µm inner diameter, 0.2 µm pore size, 70% porosity, and 450 µm wall thickness was used as a support for the electro-membrane. An ES0300-0.45 power supply from Delta Electrónica (Madrid, Spain) was used to apply electric potential between the sample and the acceptor solution, and 0.25 mm diameter platinum wires from Sigma Aldrich (St. Louis, MO, USA) were used as electrodes. Samples were stirred during extraction using an IKA^®^ big-Squid magnetic stirrer from Ika-Werke (Staufen, Germany). The pH of samples was monitored using a Jenway 4330 conductivity and pH meter (Essex, UK).

Determination of Ni concentration in the aqueous solutions was carried out using a ContrAA 700 atomic absorption spectrometer from Analytik Jena (Jena, Germany) using a graphite furnace atomizer (GFAAS) equipped with transversally heated pyrolytic graphite tubes and a xenon lamp as a continuum source of radiation.

### 2.3. Electro-Membrane Extraction Procedure

**Figure 1:**
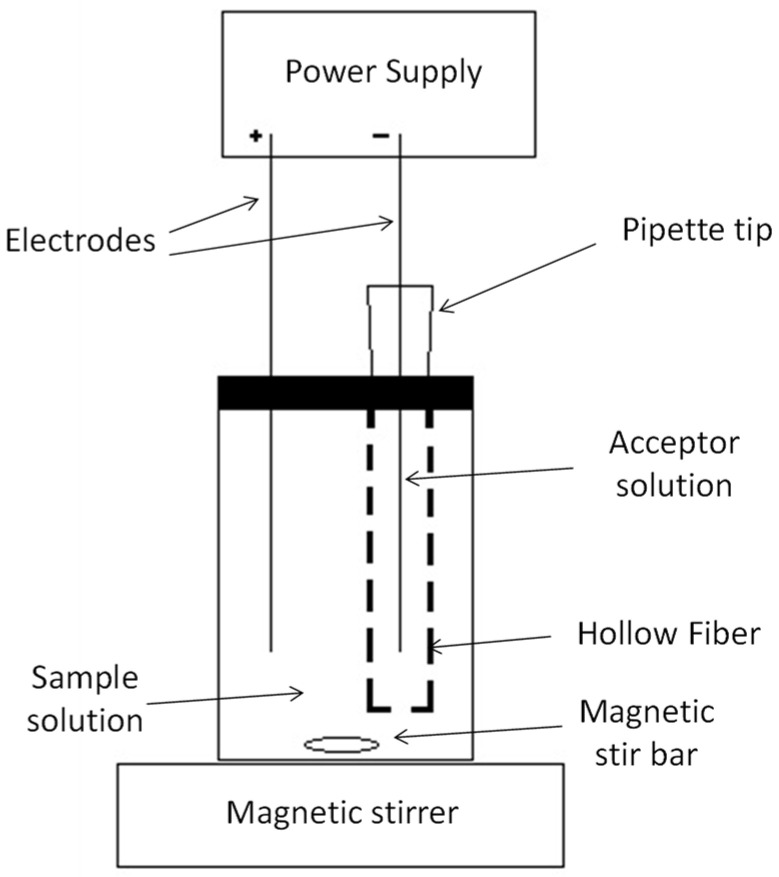
Schematic diagram of the electro-membrane system.

A piece of hollow fiber of the desired length (between 3 and 5 cm) was cut using a blade, and one of its ends was sealed using a soldering gun and flat-ended tweezers. Following this procedure, the fiber was filled up with the receiving solution (with theoretical volumes between 76 and 127 μL, respectively) and connected to a micropipette tip. Next, the fiber, while hanging on the support, was immersed in a kerosene solution containing DEHPA for 15 min to impregnate the pores in the fiber wall. After impregnation of the fiber pores with the liquid membrane, the excess organic solution in the fiber walls was rinsed with deionized water. Prior to extraction, one platinum wire was inserted in the fiber, along the whole fiber length, through the support, and a second platinum wire was placed into the sample to set up the electro-membrane system. The electrodes were connected to the power supplier, and the sample was magnetically stirred during extraction (Figure 1) [10]. In all cases, a sample volume of 50 mL was used.

Once extraction was finished, the acceptor solution was recovered and weighed before Ni quantification. In all cases, three replicates of each sample were extracted.

### 2.4. Quantification of Ni Concentration

Ni concentration in synthetic samples, as well as in the acceptor solution, was quantified using GFAAS. During dry and ash steps, 250 mL min^−1^ Ar flow was used. Ash temperature of 400 °C and atomization temperature of 2200 °C were applied for Ni determination. The analytical signal of Ni was registered for a wavelength of 232.0 nm.

Calibration curves showed to be linear up to 20 μg L^−1^. The instrumental limit of detection 0.63 μg L^−1^ was calculated as three times the standard deviation of ten calibration blanks divided by the slope of the calibration curve [23]. Each measurement was done in triplicate by using 20 µL aliquots.

### 2.5. Study of the Effect of Physical and Chemical Variables on Ni Pre-Concentration

Transport of Ni from the sample to the acceptor solution using DEHPA as a carrier takes place by coupled counter-transport of H^+^ from the acceptor solution to the sample. For this reason, HNO_3_ was used as an acceptor agent, keeping the gradient of H^+^ between the aqueous solutions as a driving force for Ni pre-concentration [21].

In order to maximize the pre-concentration of Ni from the samples into the acceptor solution, the effect of both chemical and physical variables such as DEHPA concentration in the organic solution, nitric acid concentration as acceptor solution, electric potential, fiber length, time of extraction and stirring speed were evaluated and optimized. The influence of pH in liquid microextraction of Ni with DEHPA was studied and optimized in a previous study, and thus, samples were used at pH 5.5 and buffered with 0.1M acetic acid/acetate to keep sample pH stable during extraction, minimizing the effect of H^+^ counter-transport [8].

Pre-concentration efficacy was measured as the enrichment factor (EF) of Ni in the acceptor solution, which was measured as the ratio between Ni concentration in the acceptor solution ([*Ni*]*_a_*) after extraction and the initial concentration of Ni in the source solution ([*Ni*]*_s_*) (Equation (1)).

(1) $$EF=\frac{{\left[Ni\right]}_{a}}{{\left[Ni\right]}_{s}}$$

## 3. Results and Discussion

### 3.1. Preliminary Studies

#### 3.1.1. Application of Electric Potential

As a starting point, we used the conditions the HFLPME system previously developed in the absence of electric potential, and we applied a potential varying from 15 V to 25 V to evaluate the expected enhancement of Ni transport. Initial experimental conditions were sample pH 5.5, DEHPA 0.87 M, HNO_3_ 1.86 M, extraction time 15 min, stirring rate of 500 rpm, and fiber length 4 cm. When moving from 10 V to 15 V and 25 V, values of EF of 3.8 ± 1.4, 19.3 ± 1.9, and 32.1 ± 3.9 were obtained, respectively, reinforcing the idea of exploring the improvement of the HFLPME method by using EME.

#### 3.1.2. Applicability of Different Hollow Fiber Lengths

One of the key variables in HFLPME systems is always the length of the hollow fiber used for the transport, which causes two opposite effects [24]. Thus, when the fiber length increases, the effective area of the membrane through which the transport of chemical species occurs increases as well, allowing obtaining a higher EF. Nevertheless, the volume of the internal solution also increases, causing a dilution effect and a lower EF. Additionally, the use of EME may potentiate the first of the effects described, so it is necessary to know the behavior of the new systems for different fiber lengths.

Since the manipulation of fiber systems becomes more complex when their length is shorter, the reproducibility of the recovery of the inner solution after extraction was evaluated by weighting the recovered solution in ten replicates after extraction with 3, 4, and 5 cm of fiber. In this regard, 65.9 ± 2.3 mg (RSD = 3.49%), 92.4 ± 3.1 mg (RSD = 3.35%), and 118.5 ± 3.8 mg (RSD = 3.21%) were recovered, respectively. As expected, RSD values showed lower reproducibility for shorter fibers, but this effect was almost negligible, and good reproducibility was obtained for all the fibers.

### 3.2. Optimization of Voltage

**Figure 2:**
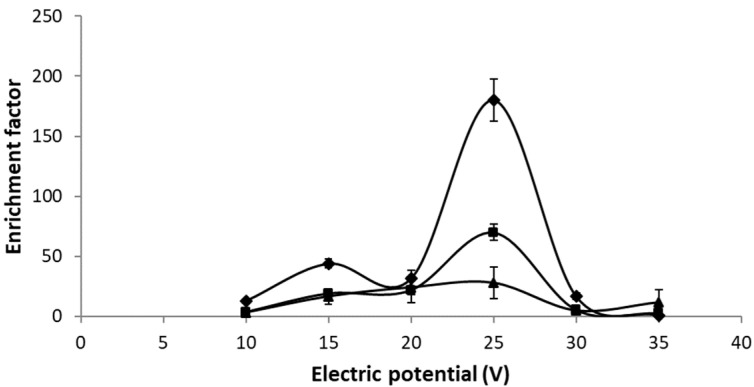
Effect of applied electric potential and fiber length on enrichment factor. Sample pH = 5.5, DEHPA 0.9 M, HNO_3_ 1.9 M, time of extraction 15 min, and stirring rate 500 rpm. Fiber length ♦ 3 cm, ■ 4 cm, ▲ 5 cm.

The effect of the electric potential was studied within the range of 10–35 V for the three different fiber lengths previously described. The results obtained are shown in Figure 2. A similar profile was observed for all the fibers, with maximum EF for 25 V. The best results were obtained for 3 cm fibers, suggesting that the effect of the dilution caused by the increase in the fiber length predominates over the greater transport due to the greater effective area.

For an electric potential greater than 25 V, the appearance of bubbles on the entire surface of the fiber was observed, causing a decrease in EF. This fact has been previously observed in other electro-membrane systems, which may cause the loss of organic solution from the fiber pores [25]. However, within the potential range selected as optimal, the extraction of nickel was efficient and reproducible, and the appearance of bubbles was not observed, suggesting that the possible variations in current intensity did not affect the performance of the EME system.

### 3.3. Optimization of Physical and Chemical Variables on the Extraction of Ni

**Figure 3:**
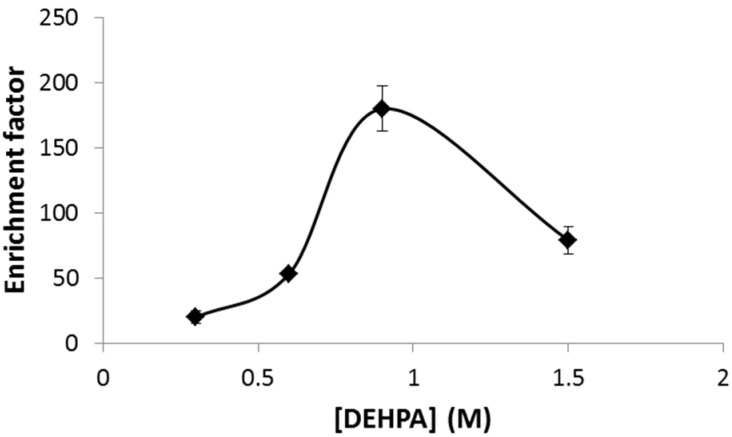
Effect of DEHPA concentration in the organic solution on the enrichment factor. Sample pH = 5.5, HNO_3_ 1.9 M. time of extraction 15 min, stirring rate 500 rpm, and electric potential 25 V.

After evaluating the effect of the electric potential, the effect of chemical variables on the transport of Ni was also studied, because it might affect the optimum variables of chemical conditions optimized for regular HFLPME and the stability of the liquid membrane in the fiber pores. In the case of DEHPA concentration in the organic solution, an increase in the enrichment factor was observed from 0.3 M until a maximum was reached at 0.9 M DEHPA (Figure 3). EF was deployed at a higher DEHPA concentration, which may be a response to an increase in the viscosity of the organic solution at high DEHPA concentrations that leads to lower diffusivity of the Ni-(DEHPA)_2_ complex [21].

**Figure 4:**
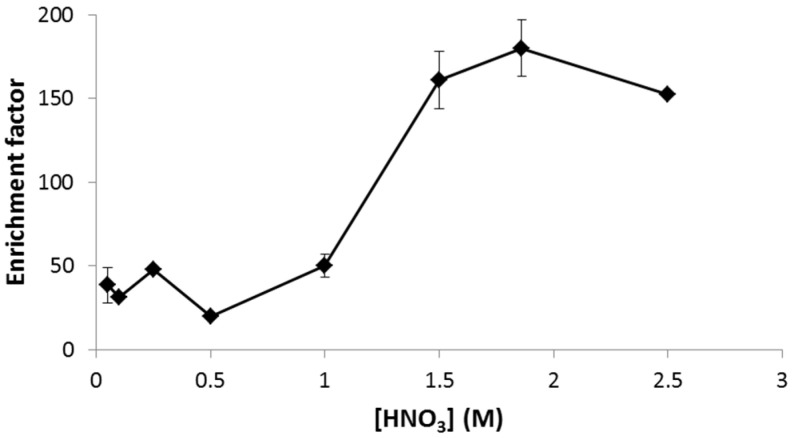
Effect of HNO_3_ concentration in the acceptor solution on the enrichment factor. Sample pH = 5.5, DEHPA 0.9 M. time of extraction 15 min, stirring rate 500 rpm, and electric potential 25 V.

Regarding conditions in the acceptor solution, the concentration of nitric acid presented a positive effect on transport from 1 M, showing a stabilization at 1.9 M HNO_3_ (Figure 4). When higher concentrations of HNO_3_ were used in the acceptor solution, a decrease in EF was observed. This could be explained by a degradation of the organic solution due to excessively acidic conditions in the acceptor solution [8].

**Figure 5:**
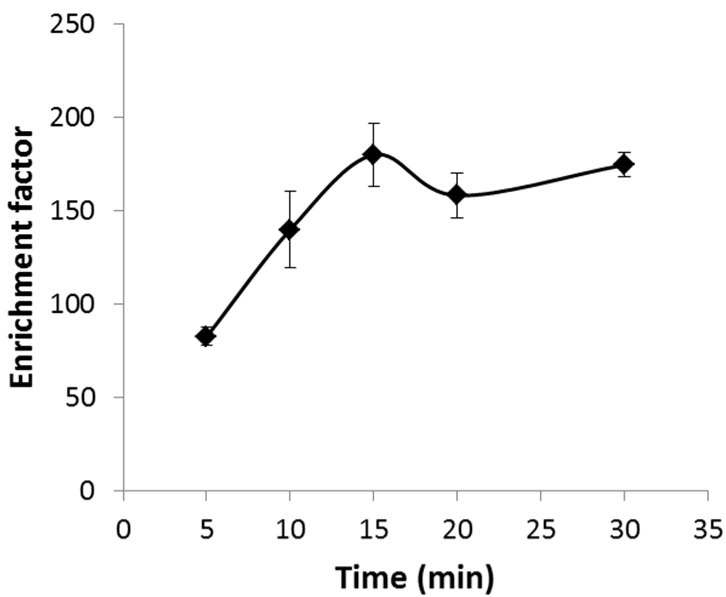
Effect of time of extraction on the enrichment factor. Sample pH = 5.5, DEHPA 0.9 M, HNO_3_ 1.9 M stirring rate 500 rpm, and electric potential 25 V.

Time of extraction was studied, and as can be seen in Figure 5, the transport of Ni was enhanced during the first 15 min and then remained stable, the variations being included within the range of uncertainty. If compared with non-electrically enhanced systems for liquid micro-extraction of Ni using the same capillary fiber, the time of operation is reduced by 89%, yielding a 125% improvement in enrichment factor [21]. Further experiments were carried out in 15 min, as the goal of the work was to keep the maxim enrichment factor using the shortest time. This extraction time is in agreement with the experimental time obtained for other electro-membrane systems [6,12,26].

**Figure 6:**
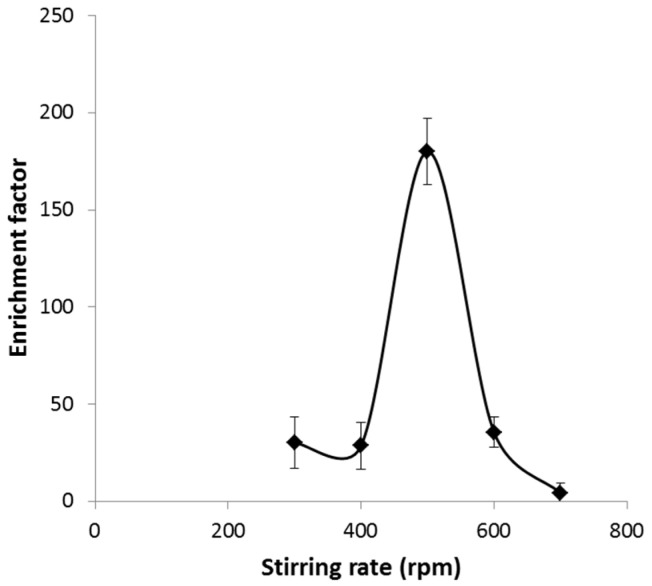
Effect of stirring rate on the enrichment factor. Sample pH = 5.5, DEHPA 0.9 M, HNO_3_ 1.9 M time of extraction 15 min, and electric potential 25 V.

In the case of the stirring speed of the samples during extraction, an increase in EF was observed up to 500 rpm due to better contact of the sample with fiber pores and the corresponding reduction of the diffusion layer between the sample and the organic solution (Figure 6). However, a reduction in the efficacy for Ni pre-concentration could be seen for a higher stirring rate due to the excessive flow rate in the sample, which led to the instability of the layer and the loss of organic solution from the fiber pores [27]. This system is particularly sensitive to stirring speed in comparison with other HFLPME systems, probably due to the existence of the electric field, which could affect the stability of the organic solution in the fiber pores [25].

Results show that when the electro-membrane systems are used, the electric potential does not affect the optimum values of the other chemical variables involved in the process, such as the concentration of DEHPA in the fiber pores or the HNO_3_ in the receiving solution. However, it dramatically accelerates the flux of Ni ions from the sample to the acceptor, leading to a higher enrichment factor in shorter times, improving the applicability of the liquid micro-extraction for Ni analysis.

In summary, the conditions that were used for the application of the electro-membrane extraction for the determination of Ni in seawater samples were: sample pH 5.5, buffer concentration 0.1 M, fiber length 3 cm, electric potential 25 V, DEHPA 0.9 M in the organic solution, HNO_3_ 1.9 M in the acceptor solution, time of extraction 15 min, and stirring rate 500 rpm, for an EF of 180 ± 17.

### 3.4. Application of DEHPA-Based Electro-Membrane for Ni Determination in Seawater Samples

Before applying the proposed electro-membrane for the determination of Ni to seawater samples, the effect of the saline matrix on the enrichment factor was evaluated. DEHPA presents a high affinity for Ni^2+^ and other divalent ions. Among the main divalent ions present in seawater, Mg^2+^ and Ca^2+^ can compete with Ni^2+^ to be transported into the acceptor solution, so a reduction of EF can be expected in real seawater samples in comparison with deionized water solutions [26]. In order to quantify the influence of the seawater matrix on Ni pre-concentration, synthetic seawater samples were prepared following the protocol described by Grashoff et al. [22] and spiked with 0.1 μg L^−1^ Ni. These samples were extracted under optimum electro-membrane conditions. The seawater matrix is known to affect the extraction of Ni due to the competence of major cations such as Ca^2+^ and Mg^2+^. We evaluated and quantified this effect, resulting in an EF of 27 ± 2 in the case of the seawater matrix, which is enough to measure Ni concentration in real seawater samples.

A series of blank experiments using synthetic seawater were carried out to calculate the methodological limit of detection (LOD). In this case, the limit of detection, calculated as the average of 10 blank measurements plus three times their standard deviation, was 23.3 ng L^−1^. Additionally, as a measurement of method precision, the intra-day relative standard deviation calculated from 10 replicates of the same sample was 5.1%, and the inter-day relative standard deviation measured in a period of 30 days was 15%. Additionally, the effect of Ni concentration in the sample was studied to establish the working range, and no dependence on EF was observed between 0.023 and 100 μg L^−1^ Ni.

The certified reference material BCR-403 from the IRMM of the Joint Research Center collected in the North Sea was analyzed using the proposed electro-membrane, and the result was compared, using a *t-*test, with the certified value [23]. Before the application of EME, the sample pH was adjusted to 5.5 using microvolumes of NaOH. The analyzed concentration was 0.29 ± 0.03 μg L^−1^, and the certified value was 0.26 ± 0.02 μg L^−1^ resulting in a *t* value of 1.79 (n = 3, α = 0.05, t_c_ = 3.18). In consequence, the electrically enhanced extraction of Ni in seawater can be used for the reliable determination of Ni in seawater samples.

Finally, the analytical features of previously existing hollow fiber liquid micro-extraction methods for Ni analysis in seawater were compared with the proposed EME (Table 1). Despite similar enrichment factors having been achieved by different methods, the electro-membrane extraction presented in this work offers accurate results for seawater samples with a significant reduction of experimental time.

## 4. Conclusions

The results provided by the proposed EME system for Ni pre-concentration improve the EF of existing liquid phase micro-extraction systems with a considerable reduction in experimental time. In this particular case, a reduction of experimental time by 89%, increasing EF of 125%, was obtained in comparison with HFLPME in the absence of an electric potential. The proposed system offers a reliable methodology for the analysis of Ni at trace level in seawater that improves the immediacy of the results. In conclusion, electro-membrane extraction can be considered an alternative to the existing time-consuming methodologies for sample preparation in the determination of metals in seawater.

## Figures and Tables

**Table 1:** Comparison of operational conditions of the proposed method and other liquid micro-extraction methods for determination of Ni in seawater.

Method	LOD (ng L^−1^)	LR (μg L^−1^)	EF	TE (min)	Ref.
HFLPME	10	--	200	120	[28]
HFLPME	170	100	536	60	[8]
SBME	44	1000	81	138	[21]
EME	23.3	100	180	15	This method

HFLPME: Hollow fiber liquid phase microextraction, SBME: Solvent bar microextraction; EME: Electro-membrane extraction; LOD: limit of detection; LR: linear range; EF: enrichment factor; TE: time of extraction.

## Data Availability

Not applicable.
